# Identification and characterization of a novel group of legume-specific, Golgi apparatus-localized WRKY and Exo70 proteins from soybean

**DOI:** 10.1093/jxb/erv104

**Published:** 2015-03-24

**Authors:** Yingjun Chi, Yan Yang, Guiping Li, Fei Wang, Baofang Fan, Zhixiang Chen

**Affiliations:** ^1^Department of Horticulture, Zijingang Campus, 866 Yuhangtang Road, Zhejiang University, Hangzhou, 310058, China; ^2^Department of Botany and Plant Pathology, 915W. State Street, Purdue University, West Lafayette, IN 47907, USA

**Keywords:** Exo70, Golgi apparatus, legumes, soybean (*Glycine max*), transmembrane, vesicle trafficking, WRKY protein.

## Abstract

Many plant genes belong to families that arise from extensive proliferation and diversification allowing the evolution of functionally new proteins. Here we report the characterization of a group of proteins evolved from WRKY and exocyst complex subunit Exo70 proteins through fusion with a novel transmembrane (TM) domain in soybean (*Glycine max*). From the soybean genome, we identified a novel WRKY-related protein (GmWRP1) that contains a WRKY domain with no binding activity for W-box sequences. GFP fusion revealed that GmWRP1 was targeted to the Golgi apparatus through its N-terminal TM domain. Similar Golgi-targeting TM domains were also identified in members of a new subfamily of Exo70J proteins involved in vesicle trafficking. The novel TM domains are structurally most similar to the endosomal cytochrome b561 from birds and close homologues of GmWRP1 and GmEx070J proteins with the novel TM domain have only been identified in legumes. Transient expression of some GmExo70J proteins or the Golgi-targeting TM domain in tobacco altered the subcellular structures labelled by a fluorescent Golgi marker. *GmWRP1* transcripts were detected at high levels in roots, flowers, pods, and seeds, and the expression levels of *GmWRP1* and *GmExo70J* genes were elevated with increased age in leaves. The legume-specific, Golgi apparatus-localized GmWRP1 and GmExo70J proteins are probably involved in Golgi-mediated vesicle trafficking of biological molecules that are uniquely important to legumes.

## Highlight

Soybean contains a WRKY-related (GmWRP1) and GmExo70J protein subfamily found only in legumes. GmWRP1 and some GmExo70J proteins are targeted to the Golgi apparatus through a novel N-terminal transmembrane domain.

## Introduction

The birth of new genes with novel functions is a key contributor to adaptive evolution of organisms (Kaessmann, 2010). A central process in the origin of new genes for biological evolution is gene duplication, which leads to the evolution of gene families (Kaessmann, 2010). In *Arabidopsis*, despite the small genome size, the majority of its genes belong to families with two or more members (Initiative, 2000). As an ancient tetraploid, some of the gene families in *Arabidopsis* are attributed to large-scale genomic duplication events that occurred 100–200 million years ago (Blanc *et al.*, 2000; Vision *et al.*, 2000; Blanc *et al.*, 2003). Other gene families in *Arabidopsis* have resulted from independent, small-scale gene duplication events. Some of these small-scale gene duplications produced tandem repeats of related genes while others generated dispersed gene families. As a result, many plant genes belong to large families or superfamilies with a large number of family members. Members of plant gene families often display extensive sequence similarity and their diversification usually results from the accumulation of subtle genetic modifications that can lead to differences in their structures and functions. Functional analysis has repeatedly revealed that gene family members can have redundant functions, probably reflecting those performed by their pre-existing ancestral genes in certain biological processes, but have distinct, cooperative, or antagonistic roles in other biological processes. Another major route of protein evolution is through gene fusion leading to the formation of multi-domain proteins. Naturally occurring fusion genes are often created through chromosomal translocation, which replaces the terminal exons of one gene with intact exons from a second gene (Kaessmann, 2010). Many important cancer-promoting oncogenes are fusion genes produced in this way (Aman, 2005*a*, b; Teixeira, 2006; Bartek *et al.*, 2010).

Legumes include many important crop plants cultivated worldwide, with most of them capable of fixing atmospheric nitrogen through intimate symbiosis with microorganisms. Among cultivated legumes, soybean (*Glycine max*) is a particularly important crop as a predominant plant source of proteins, cooking oil, and other nutrients beneficial to human health. The genomes of a number of legumes including soybean have been completely sequenced. Soybean is an ancient polyploid with two genome duplications that occurred at approximately 59 and 13 million years ago, followed by gene loss, diversification, and chromosome rearrangements (Schmutz *et al.*, 2010). The well annotated genome sequences from a number of legumes will greatly facilitate identification of the molecular and genetic basis of important legume traits using genetic, molecular, and genomic tools. It is clear that many of the genes and pathways involved in important biological processes of legumes, including nodulation, isoflavonoid biosynthesis, and vesicle trafficking of storage proteins, evolved from those in other plant species (Schmutz *et al.*, 2010). In addition, legumes have novel genes that have diverged greatly from their progenitors with new functions important for legume-specific traits. For example, a previous study using BLAST algorithms identified putative legume-specific genes with no sequence homology, below a specified threshold, to sequences of non-legumes (Graham *et al.*, 2004). Identification and functional characterization of legume-specific genes could provide novel insights into the genetic and molecular basis of hallmark legume functions.

During our recent analysis of the soybean WRKY transcription factor superfamily, we discovered a WRKY-related protein (GmWRP1, Glyma14g37960) that appears to be derived from the fusion of the N-terminal WRKY domain of a group I WRKY protein with a novel N-terminal region that is predicted to be a five-pass transmembrane (TM) domain. As transcription factors, WRKY proteins are mostly soluble and, in rare situations where they are associated with cellular membranes for regulatory purposes, they do so through interactions with membrane-associated proteins (Shang *et al.*, 2010). In the present study, we report that unlike typical WRKY proteins, GmWRP1 is unable to bind the TTGACC W-box sequences and is exclusively targeted to the Golgi complex through its N-terminal TM domain. Similar Golgi-targeting TM domains are also identified in a number of soybean proteins, including members of a subfamily of Exo70J proteins phylogenetically distinct from the nine subclades of Exo70 proteins previously identified from other plants (Synek *et al.*, 2006; Chong *et al.*, 2010; Li *et al.*, 2010). The Golgi-targeting TM domains are structurally most similar to the endosomal cytochrome (cyt) b561 from birds, suggesting horizontal gene transfer as a potential mechanism for the evolution of the novel genes. Intriguingly, homologues of soybean GmWRP1, GmEx070J, or other proteins with the novel Golgi-targeting TM domains, are identified only in legumes and not in other plant species. Transient overexpression of some of the legume-specific GmExo70 proteins or the Golgi-targeting TM domain in tobacco leaves drastically changed the subcellular structures labelled by a fluorescent Golgi marker. These results strongly suggest that the novel legume-specific *GmWRP1* and *GmExo70J* genes resulted from the evolution of two highly expanded plant gene families with possible new roles in vesicle trafficking of biological molecules highly important for legumes.

## Materials and methods

### Plant materials and growth conditions

Soybean (*Glycine max* cv. ‘Williams 82’), *Arabidopsis*, and tobacco (*Nicotiana benthamiana*) plants were grown in a greenhouse or growth room at 25°C with a photoperiod of 12h.

### Production of recombinant protein and EMSA

To generate GmWRP1 recombinant protein, its full-length cDNA was PCR-amplified using gene-specific primers (5′-AGCGGA TCCATGCCTTTCCGAAATTCCAATAT-3′ and 5′-AGCCTCG AGTCATATTTCATTTGGATATTGAGGAG-3′), cloned into pET32a (Novagen, San Diego, CA, USA), and transformed into *Escherichia coli* strain BL21 (DE3). The chimeric pET32a-AtWRKY33 construct has been described previously (Zheng *et al.*, 2006). Induction of protein expression and purification of recombinant His-tagged proteins were performed according to the protocol provided by Novagen. Labelling of double-stranded synthetic oligonucleotides using the Klenow fragment of DNA polymerase I and sequence-specific DNA binding with EMSA were performed essentially as described previously (Zheng *et al.*, 2006).

### Subcellular localization

DNA fragments for full-length or truncated GmWRP1 and GmExo70J proteins were PCR-amplified using gene-specific primers listed in Supplementary Table S1 and fused to the *GFP* gene (Supplementary Figure S1) behind the CaMV *35S* promoter in a modified pCAMBIA1300 plant transformation vector. Correct sequences and fusion of the constructs were confirmed by DNA sequencing. *Agrobacterium* cells containing the GFP fusion constructs were co-infiltrated with the ST-mRFP Golgi marker or another endomembrane marker into *Nicotiana benthamiana* leaves. Two days after infiltration, imaging of co-expressed GFP, mRFP, and mCherry signals was performed with standard confocal laser microscopy (Wang *et al.*, 2014). The levels of GFP fusion proteins in infiltrated tobacco leaves were determined with western blotting using an anti-GFP antibody as described previously (Wang *et al.*, 2014).

Identification and phylogenetic analysis of soybean Exo70 proteins

Published *Arabidopsis* AtExo70 protein sequences were used in BLASTp searches for GmExo70 proteins in the soybean genome (http://www.phytozome.net, Glycine max v1.1). All final data sets were downloaded in June 2014. The Pfam database was employed to ascertain whether the candidate proteins contained features typical of Exo70 proteins. Phylogenetic trees based on complete amino acid sequences of Exo70 proteins from soybean, *Arabidopsis*, and other species were constructed using MAFFT (Katoh and Standley, 2013) and FastTree (Price *et al.*, 2009) by the maximum likelihood method with 1000 bootstraps. FigTree (http://tree.bio.ed.ac.uk/software/figtree/) was used for tree plotting.

### Analysis of gene expression using qRT-PCR

Soybean tissue samples were lyophilized and stored at –80°C until use. Total RNA was isolated from soybean tissues using the Trizol reagent according to the supplier’s instructions. Extracted RNA was treated with DNase to remove contaminating DNA and reverse transcribed using the ReverTran Ace^®^ qPCR RT kit (Toyobo) for reverse transcriptase-PCR. qRT-PCR was performed with a StepOnePlus^TM^ Real-Time PCR System (ABI). PCRs were performed using the SYBR^®^ Green qPCR Master Mixes (Takara) and gene-specific primers listed in Supplementary Table S2. The PCR conditions consisted of denaturation at 95°C for 1min, followed by 40 cycles of denaturation at 95°C for 15 s, and annealing and extension at 58°C for 30 s. Relative gene expression was calculated as previously described (Livak and Schmittgen, 2001). The soybean actin gene (Glyma18g52780, 5′-GTGCACAATTGATGGACCAG-3′ and 5′-GCACCACCGGAGAGAAAATA-3′) was used as an internal control.

## Results

### Identification of GmWRP1, a WRKY-related protein from soybean

**Fig. 1.**
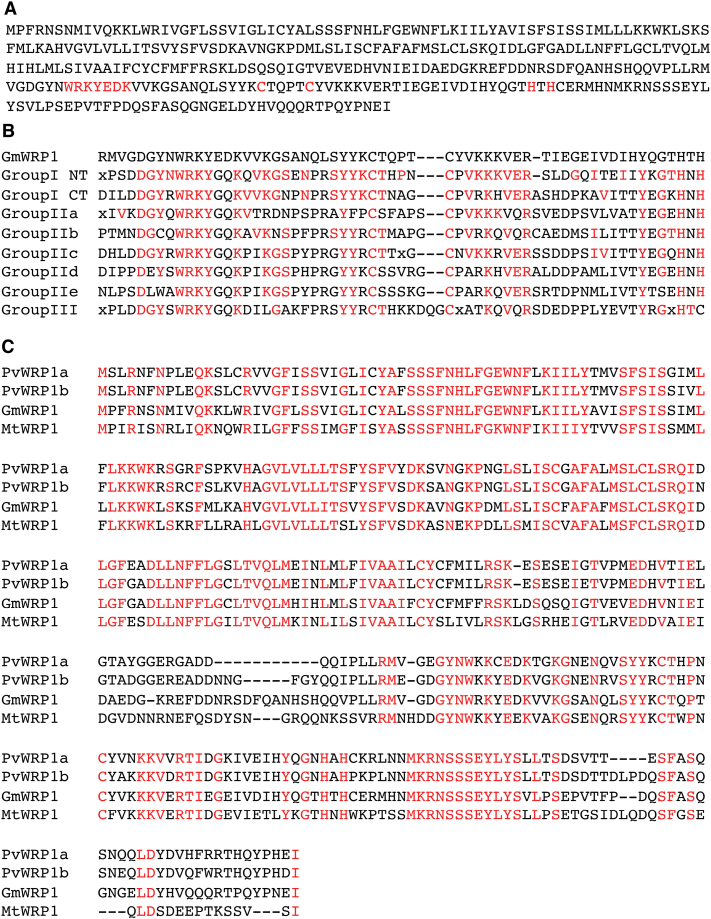
Sequence analysis of GmWRP1. (A) Amino acid sequence of GmWRP1. The hallmark amino acid residues for the WRKY domain are shown in red. (B) Alignment of the GmWRP1 WRKY domain sequence with the consensus sequences of the WRKY domains of seven WRKY subfamilies, including the N-terminal (NT) and C-terminal (CT) WRKY domains of group I WRKY proteins (Rushton *et al.*, 2010). The amino acid residues in the consensus sequences of the WRKY domains identical to those in GmWRP1 are shown in red. (C) Close homologues of GmWRP1 in legume plants. Amino acid sequences of the WRP1 proteins from *P. vulgaris* (PvWRP1a and PvWRP1b), *G. max* (GmWRP1), and *M. truncatula* (MtWRP1) are from their annotated genomes, and fully conserved amino acid residues are shown in red.

WRKY transcription factors are encoded by large gene families with more than 70 members in *Arabidopsis* (Rushton *et al.*, 2010). Based on the number and amino acid sequence of the WRKY zinc-finger motifs, the WRKY family was originally divided into three groups. Group I WRKY proteins contains two C2H2 zinc-finger motifs, while groups II and III contain one C2H2 and one C2HC zinc-finger motif, respectively (Eulgem *et al.*, 2000). Additional phylogenetic analyses, however, have shown that the group II proteins can be further divided into groups IIa–e (Zhang and Wang, 2005). Through extensive searching and sequence verification with the annotated soybean genome, we identified more than 170 WRKY genes from soybean, which is significantly more than the 133 members identified from a previously reported study with BLAST searching used *Arabidopsis* WRKY proteins as queries (Yin *et al.*, 2013), but similar to the number identified from a more recent study through genome-wide annotation (Bencke-Malato *et al.*, 2014). Phylogenetic analysis through multiple sequence alignment of the WRKY domains classifies the vast majority of soybean WRKY proteins into one of the seven groups. There were, however, a few exceptions, including Glyma14g37960 (Fig. 1A). Comparison with the consensus WRKY-domain sequences of the seven groups of WRKY proteins (Rushton *et al.*, 2010) indicated that the WRKY domain of Glyma14g37960 is most closely related to the N-terminal WRKY domain of group I WRKY proteins (Fig. 1B). However, unlike typical group I WRKY proteins with two WRKY domains, Glyma14g37960 does not contain the C-terminal WRKY domain, which is likely to be responsible for the sequence-specific DNA-binding activity of group I WRKY proteins (de Pater *et al.*, 1996; Ishiguro and Nakamura, 1994). In addition, the almost invariant WRKYGQK sequence at the N-terminus of the WRKY domain in the vast majority of WRKY proteins was changed into WRKYEDK in Glyma14g37960 (Fig. 1A and 1B). As will be described later, Glyma14g37960 contains a novel domain at its N-terminus and is localized in the Golgi apparatus. Thus Glyma14g37960 has undergone extensive structural modifications, most likely resulting in novel biological functions. Based on these observations, Glyma14g37960 was named soybean WRKY-related protein 1 (GmWRP1).

### Lack of binding activity of GmWRP1 to TTGACC W-boxes

**Fig. 2.**
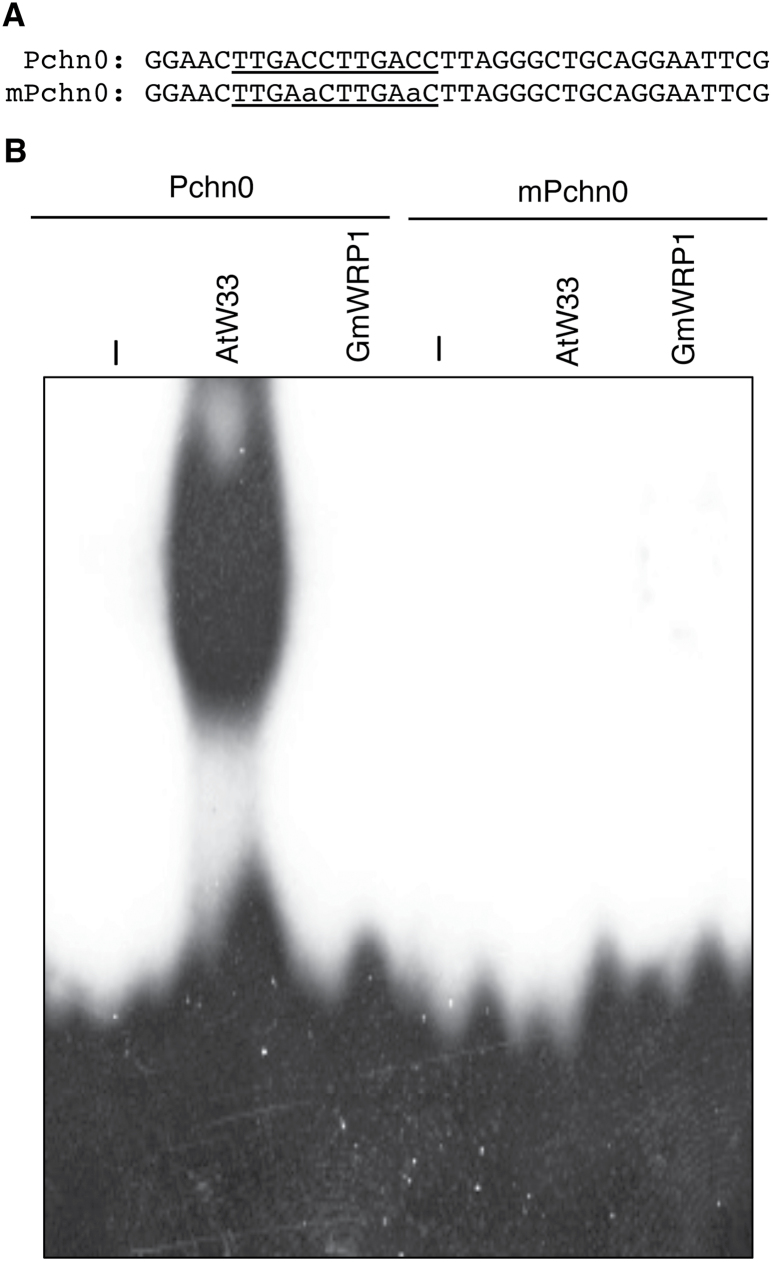
Analysis of DNA binding of GmWRP1 recombinant proteins. (A) Sequences of the Pchn0 probe, which contains two TTGACC W-box sequences, and the mPchn0 probe, which has the TTGACC sequences mutated into TTGAaC. (B) Sequence-specific binding of Pchn0 by *Arabidopsis* AtWRKY33 (AtW33) but not soybean GmWRP1 recombinant proteins. In mPchn0, changes in the TTGACC sequences to TTGAaC resulted in complete abolishment of binding by AtWRKY33.

WRKY proteins bind the TTGACC/T W-box sequences that are present in the promoters of a large number of plant genes, particularly those involved in plant responses to biotic and abiotic stresses (Dong *et al.*, 2003; Eulgem, 2006; Rushton *et al.*, 2010). To determine whether GmWRP1 retains the most important molecular activity of WRKY proteins, we examined the sequence-specific DNA-binding activity of recombinant GmWRP1 using electrophoretic mobility shift assay (EMSA). As a positive control, we included *Arabidopsis* AtWRKY33, a group I WRKY protein that is known to bind W-boxes in a sequence-specific manner (Zheng *et al.*, 2006; Lai *et al.*, 2011). When recombinant AtWRKY33 proteins were incubated with a labelled Pchn0 probe, which contains two TTGACC W-box sequences (Yang *et al.*, 1999), we detected a strong DNA-binding activity by AtWRKY33 based on the appearance of an intense band with reduced mobility (Fig. 2). To determine whether AtWRKY33 specifically recognized the TTGACC W-box sequences, we also tested a mutant probe (mPchm) in which the two TTGACC sequences were mutated into TTGAAC. As shown in Fig. 2, the mutant probe was not recognized by recombinant AtWRKY33. On the other hand, when recombinant GmWRP1 proteins were incubated with Pchn or mPchn0, no retarded band was detected by EMSA. These results indicate that GmWRP1 is unable to recognize W-box sequences.

### Localization of GmWRP1 to the Golgi apparatus

**Fig. 3.**
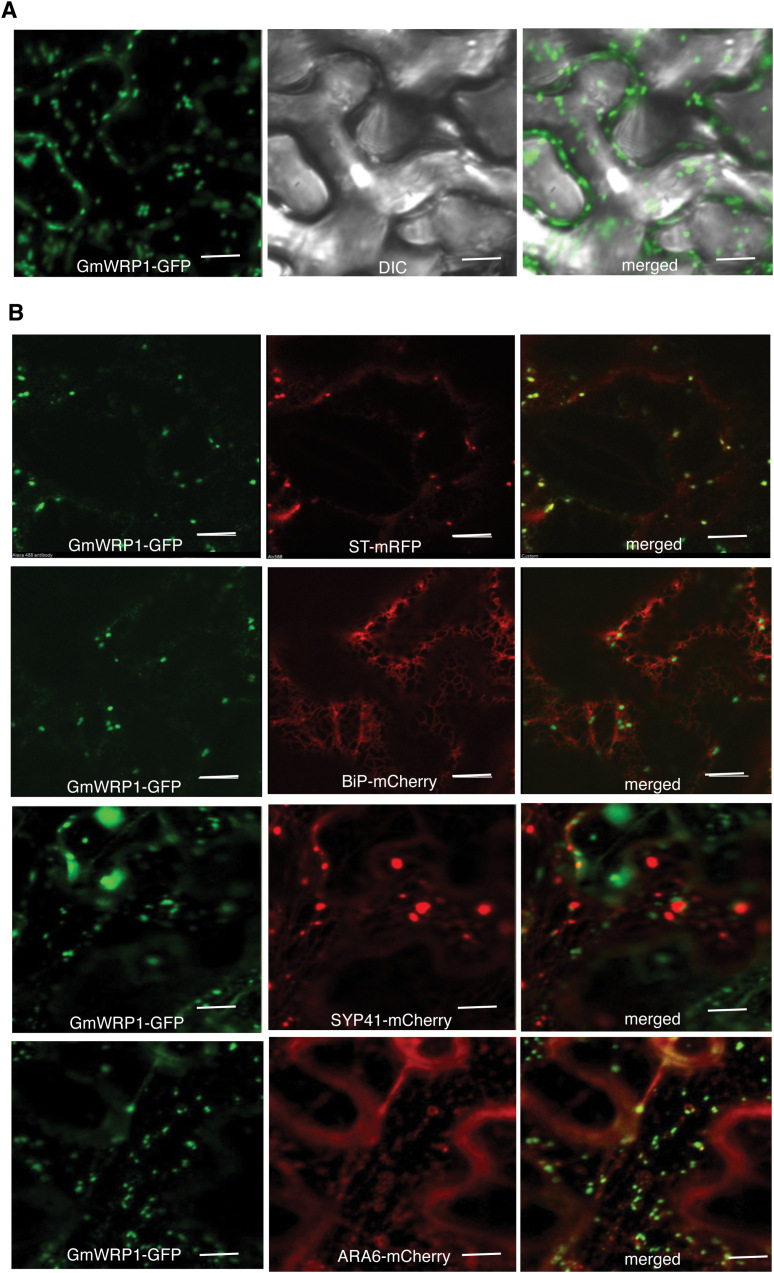
Localization of GmWRP1 in the Golgi apparatus. The GmWRP1–GFP fusion gene was co-expressed with Golgi apparatus marker ST-mRFP, ER marker BIP-mCherry-KDEL, TGN marker SYP41-mCherry, or MVB marker ARA6-mCherry in *N. benthamiana*. The GFP, mRFP, DIC, and merged images are shown. A majority of GmWRP1–GFP fluorescence signals were also labelled by ST-mRFP Golgi marker signals. However, few of the GmWRP1–GFP punctate fluorescence signals were labelled by BIP-mCherry-KDEL ER marker, SYP41-mCherry TGN marker, or ARA6-mCherry MVB marker signals. Bar, 10 µm.

The lack of GmWRP1 activity to bind W-box sequences raises the possibility of novel functions of the WRKY-related protein in soybean. To examine this possibility, we first analysed the subcellular localization of GmWRP1. We generated a *GmWRP1–GFP* fusion gene (Supplementary Figure S1) and transiently expressed it in *N. benthamiana.* Interestingly, in tobacco leaf epidermal cells expressing *GmWRP1–GFP*, when viewed by confocal fluorescence microscopy we observed punctate fluorescent signals in the cytoplasm (Fig. 3A). To identify the subcellular structures relating to the punctate signals, we co-expressed GmWRP1–GFP with an mRFP fused with the signal anchor of a rat sialyl transferase (ST-mRFP), a fluorescent Golgi apparatus marker (Kim *et al.*, 2001). As shown in Fig. 3B, the co-localization analysis revealed that >90% of the GmWRP1–GFP punctate signals were labelled by ST-mRFP. By contrast, co-localization analysis between GmWRP1–GFP and a BIP-mCherry-HDEL fluorescent marker of endoplasmic reticulum showed little overlapping of labelled subcellular structures (Fig. 3B). We also co-expressed GmWRP1–GFP with markers of the *trans*-Golgi network (TGN) (Syp41-mCherry) and multivesicular bodies (MVB) (Ara6-mCherry), both of which also exhibit punctate fluorescence signals when expressed in plant cells (Dettmer *et al.*, 2006; Ebine *et al.*, 2011; Lin *et al.*, 2014). Again we observed little overlapping of the GmWRP1–GFP signals with those of Syp41-mCherry or ARA6-mCherry (Fig. 3B). These results indicated that GmWRP1 is primarily localized in Golgi complexes of plant cells.

### Identification of a distinct subfamily of GmExo70 proteins related to GmWRP1

Searching the annotated soybean genome revealed no other soybean WRKY genes encoding proteins highly similar to GmWRP1 beyond the conserved WRKY domain. Searching the *Arabidopsis*, rice, and poplar genomes also failed to find close structural homologues of GmWRP1. However, a single protein from *Medicago truncatula* and two from *Phaseolus vulgaris* identified from their sequenced genomes have extensive sequence similarity to GmWRP1 throughout their entire length, not only in the C-terminal WRKY domain but also in the N-terminal region (Fig. 1C). BLAST searches of all non-redundant GenBank coding sequences also identified highly similar homologues of GmWRP1 in chickpea (*Cicer arietinum*). Thus, WRP1 appears to be a highly conserved protein in legumes.

**Fig. 4.**
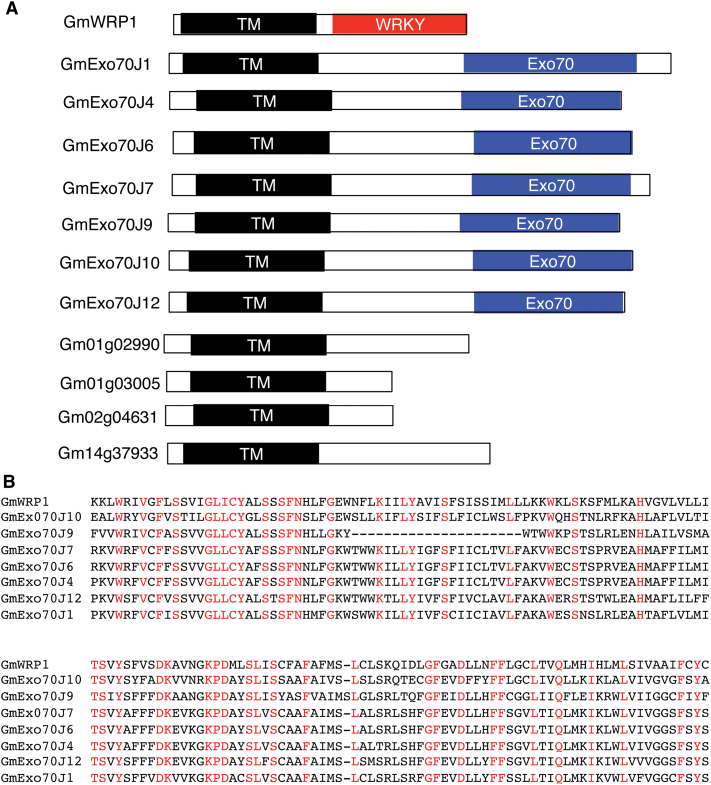
Soybean proteins containing N-terminal TM domains. (A) Diagrams of soybean proteins containing N-terminal transmembrane (TM) domains with or without an additional WRKY or Exo70 domain at the C-terminus. (B) Alignment of the amino acid residues of the N-terminal TM domain of GmWRP1 with those of the TM domains from GmExo70J proteins. Fully conserved amino acid residues are show in red.

**Fig. 5.**
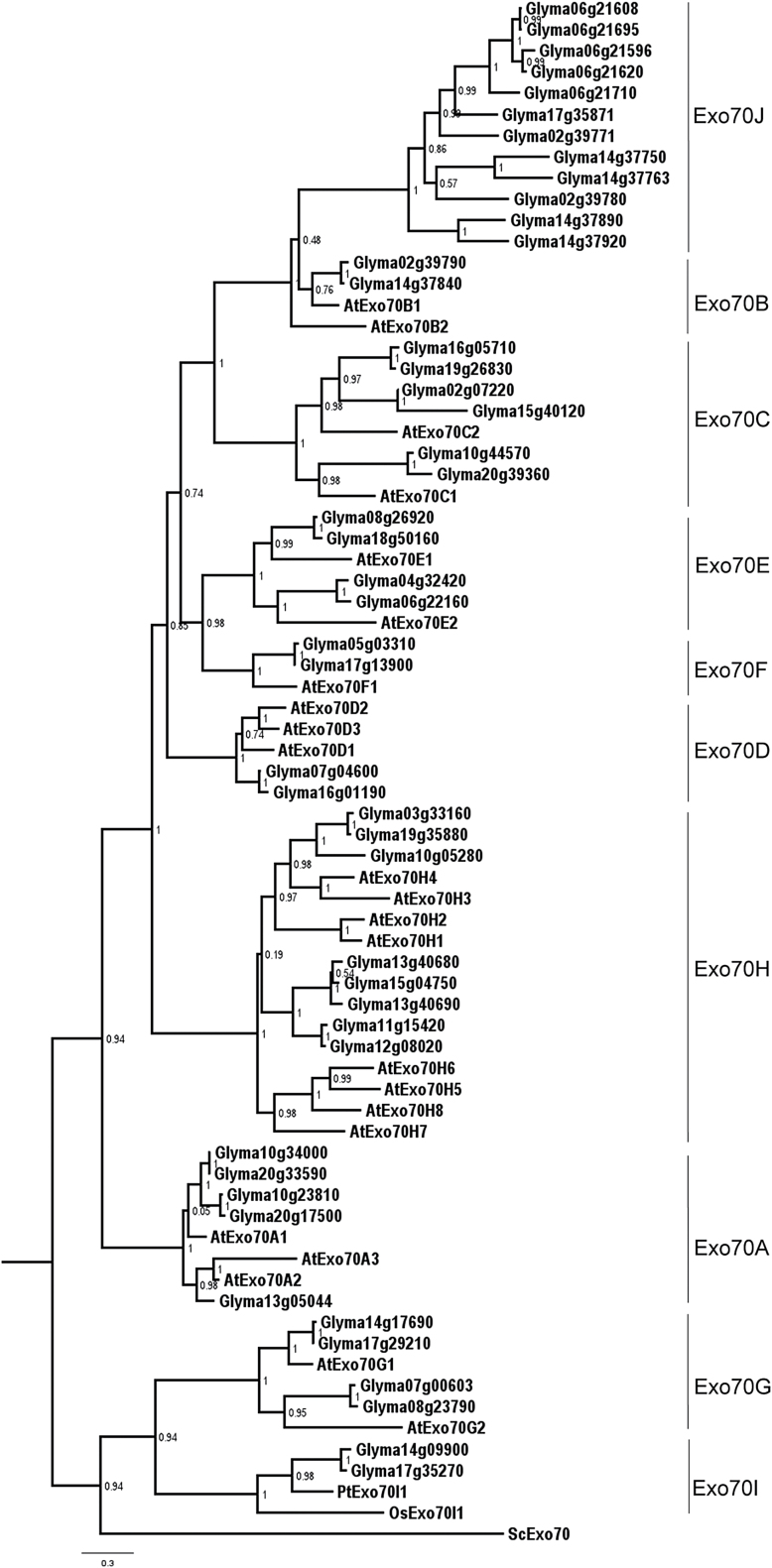
Phylogenetic analysis of full-length plant Exo70 proteins. The phylogenetic tree was inferred using the maximum likelihood methods from 47 soybean GmExo70 proteins, 23 *Arabidopsis* AtExo70 proteins, two rice Exo70I proteins, and both poplar and yeast Exo70. Bootstrap values from 1000 replicates were used to assess the robustness of the tree. Ten subclades of plant Exo70 proteins are indicated.

Searching the annotated soybean genome also revealed a group of about 10 proteins whose N-terminal regions are highly similar to the N-terminal region of GmWRP1 (Fig. 4). Among the 10 proteins, seven contain a C-terminal region highly similar to the exocyst complex subunit Exo70 (Fig. 4A). Exocyst is a conserved complex of eight proteins (SEC3, SEC5, SEC6, SEC8, SEC10, Sec15, Exo70, and Exo84) that tethers Golgi-derived vesicles to the targeted plasma membrane during exocytosis (Finger *et al.*, 1998; Guo *et al*., 1999*a*, *b*; Boyd *et al.*, 2004; Samuel *et al.*, 2009). Unlike other exocyst subunits, which undergo no or very limited gene expansion, the genes for Exo70 have expanded greatly in plants, with 23 in *Arabidopsis* (Synek *et al.*, 2006; Chong *et al.*, 2010), in contrast to a single Exo70 gene in yeast and many animals. Phylogenetically, plant EXO70 proteins can be divided into three clades (Exo70.1 to Exo70.3) and nine subclades (Exo70A to Exo70I) (Synek *et al.*, 2006; Chong *et al.*, 2010). The reported phylogenetic analysis of plant Exo70 proteins, however, did not include those from soybean or other legumes and, therefore, it is unclear whether the identified soybean Exo70 proteins belong to one or more of the nine identified subclades. Using keyword and BLAST searches using Exo70 protein sequences from *Arabidopsis* as queries, we identified 56 soybean *Exo70* genes from sequenced soybean genome. Among the 56 putative *Exo70* genes, two encode proteins containing no Exo70 domain and seven other genes encode proteins with only short Exo70 motifs. Thus, there are at least 47 genes in soybean that encode full-length Exo70 proteins. A maximum likelihood phylogenetic tree was then generated with the 23 Exo70 proteins from *Arabidopsis*, which form eight subclades (Exo70A to Exo70H), two Exo70I subclade proteins from rice and poplar, and 47 soybean Exo70 proteins. As previously reported (Synek *et al.*, 2006; Chong *et al.*, 2010), the *Arabidopsis*, rice, and poplar Exo70 proteins were grouped into nine subclades (Exo70A to Exo70I) (Fig. 5). In addition, 35 of the 47 soybean Exo70 proteins were also grouped into the same nine subclades as *Arabidopsis*, rice, and poplar Exo70 proteins (Fig. 5). The remaining 12 soybean Exo870 proteins, however, did not belong to any of the nine established subclades; instead they formed a distinct subclade (Fig. 5). Phylogenetic analysis using the Exo70 domain sequences of the same set of *Arabidopsis*, rice, poplar, and soybean Exo70 proteins also grouped the 12 soybean Exo70 proteins into a distinct subclade (Supplementary Figure S2), which we have named the Exo70J subclade. Accordingly, the 12 soybean Exo70J proteins are named GmExo70J1 to GmExo70J12 (Table 1).

**Table 1.** Soybean GmExo70J proteins

Name	Gene identifier	No. of amino acids	N-terminal TM domain	Introns within CD
GmExo70J1	Glyma02g39771	736	+	0
GmExo70J2	Glyma02g39780	537	–	0
GmExo70J3	Glyma06g21596	411	–	0
GmExo70J4	Glyma06g21608	639	+	1
GmExo70J5	Glyma06g21620	410	–	0
GmExo70J6	Glyma06g21695	657	+	0
GmExo70J7	Glyma06g21710	684	+	0
GmExo70J8	Glyma14g37750	552	–	0
GmExo70J9	Glyma14g37763	628	+	4
GmExo70J10	Glyma14g37890	676	+	0
GmExo70J11	Glyma14g37920	459	–	0
GmExo70J12	Glyma17g35871	648	+	0

A survey of the 12 GmExo70J proteins revealed a number of interesting features. First, the seven Exo70 proteins that share the highly conserved N-terminal domain with GmWPR1 all belong to this new Exo70J subclade (Table 1). Second, phylogenetically, soybean GmExo70J proteins are mostly closely related to the two GmExo70B proteins (Glym02g39790 and Glym14g37840), suggesting that the soybean GmExo70J subclade may have evolved from an ancestral Exo70B protein. Third, all the GmExo70J proteins are encoded by genes distributed on four chromosomes (Chr 2, 6, 14, and 17), suggesting that expansion of the genes may be associated with the two genome duplications of soybean. However, there are multiple and clustered *GmExo70J* genes on Chr 2, 6, and 14, indicating that local gene duplication also contributed to expansion of *GmExo70J* genes. Notably, the two *GmExo70B* genes are among the clustered *GmExo70J* genes on Chr 2 and 14, supporting the phylogenetic evidence that the GmExo70B and GmExo70J subclades are evolutionarily closely related.

### Subcellular localization of soybean Exo70J proteins

**Fig. 6.**
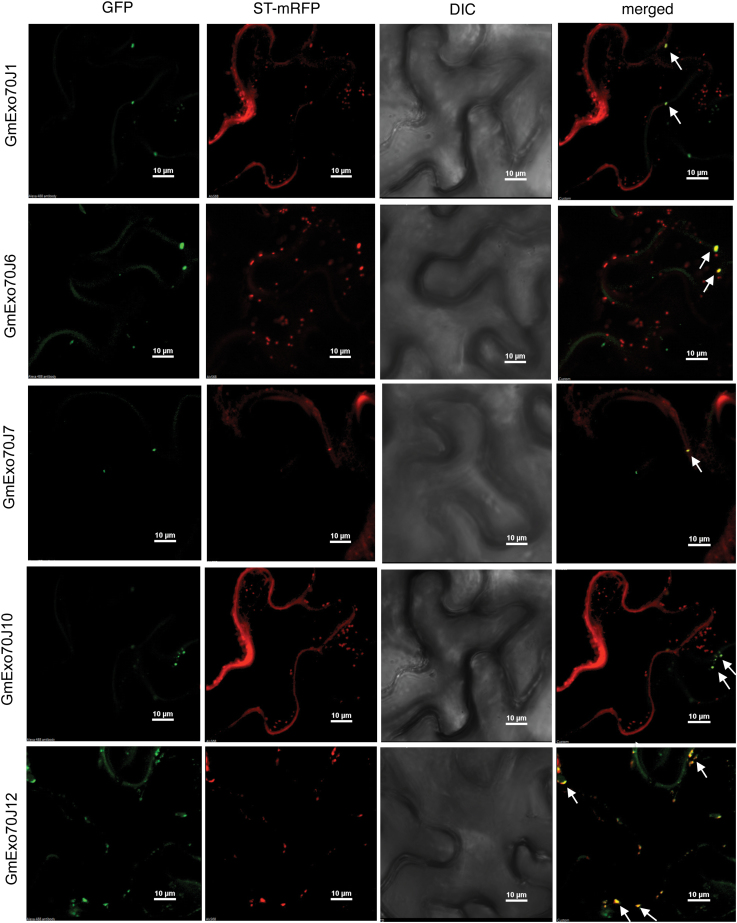
Subcellular localization of soybean GmExo70 proteins. The GmExo70J–GFP fusion genes were coexpressed with the Golgi apparatus marker gene ST-mRFP in *N. benthamiana*. The GFP, mRFP, DIC, and merged images are shown. A majority of GmExo70J–GFP punctate fluorescence signals were also labelled by the ST-RFP Golgi marker signals, with some indicated by arrows. Co-expression of GmExo70J1, GmExo70J7, and GmExo70J10 also altered the labelling patterns of the ST-RFP Golgi marker. Bar, 10 µm.

Yeast and animal Exo70 proteins are localized to the plasma membrane. However, it has been shown that some members of the expanded plant Exo70 family are localized to other subcellular compartments (Pecenkova *et al.*, 2011; Kulich *et al.*, 2013; Stegmann *et al.*, 2013). Since some of the GmExo70J proteins shared a highly similar N-terminal region with the Golgi-localized GmWRP1, we also analysed the subcellular localization of these GmExo70J proteins. For this purpose, we tagged GFP to the C-terminus of five full-length GmExo70J proteins (GmExo70J1, 6, 7, 10, and 12), which all contain the legume-specific N-terminal domain as GmWRP1 (Fig. 4; Supplementary Figure S1). These GFP fusion constructs were transiently co-expressed in *N. benthamiana* with the ST-mRFP Golgi marker. In tobacco leaf epidermal cells expressing GFP fusion proteins, we observed relatively much lower levels of fluorescence from expression of the Exo70J fusion proteins when compared to those of GmWRP1–GFP or ST-mRFP marker. Western blotting using an anti-GFP antibody detected GFP fusion proteins with expected sizes in the tobacco leaves infiltrated with the constructs (Supplementary Figure S3). Consistent with the intensities of GFP-fluorescent signals, levels of the five GmExo70J–GFP fusion proteins detected by western blotting were much lower than those of GmWRP1–GFP (Supplementary Figure S3). The low levels of GmExo70J–GFP proteins were apparently not caused by co-expressed ST-mRFP maker because individual expression of the *GmExo70J–GFP* genes in the absence of the Golgi marker also generated similarly low levels of the proteins (data not shown). However, the detected fluorescent signals for the GmExo70J–GFP fusion proteins mostly displayed punctate structures in the cytoplasm that were also labelled by the ST-mRFP Golgi marker when viewed by confocal fluorescence microscopy (Fig. 6). These observations indicated that like GmWRP1, the five Exo70J proteins are targeted to the Golgi complex. Interestingly, in the tobacco epidermal cells expressing GmExo70J1, GmExo70J7, and GmExo70J10 proteins, the fluorescent signals of co-expressed ST-RFP displayed not only punctate structures but also increased levels of a discrete network (Fig. 6). Thus, transient overexpression of three of the Exo70J proteins appeared to cause altered distribution of the ST-mRFP fluorescent signals in the cells.

### Identification of the novel TM domain in GmWRP1 and GmExo70J

**Fig. 7.**
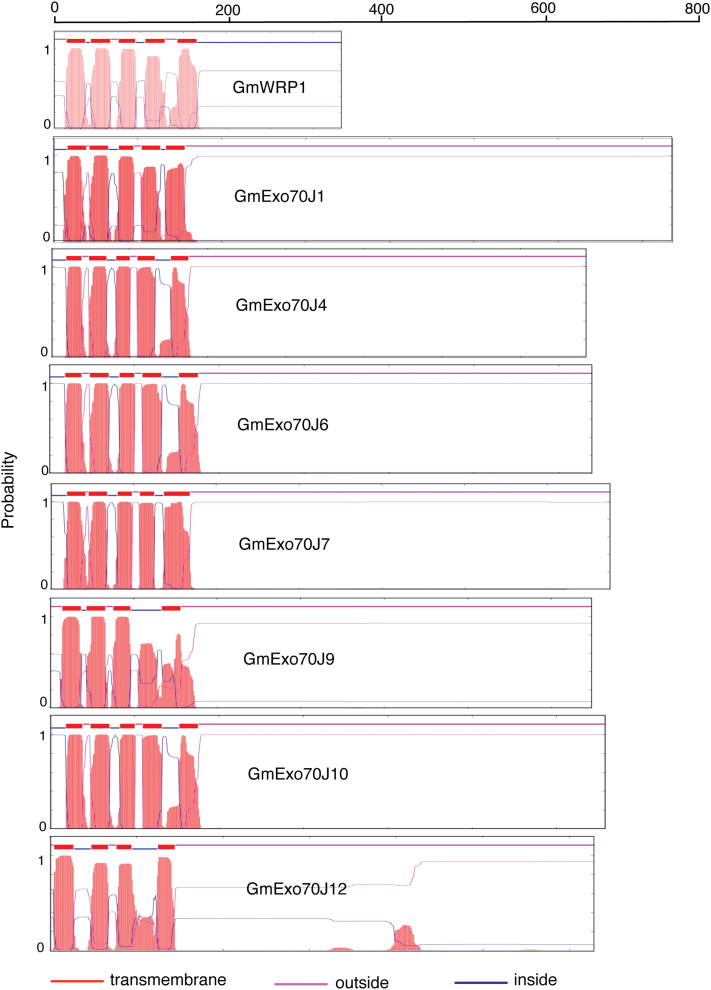
Prediction of the conserved N-terminal domain in GmWRP1 and Exo70J proteins as a five-passage TM domain. Prediction of TM helices and orientation in proteins were performed using the TMHMM method based on a hidden Markove model (http://www.cbs.dtu.dk/services/TMHMM-2.0/). The size of each protein is indicated by the number of amino acid residues shown at the top. This figure is available in colour at *JXB* online.

GmWRP1 and seven GmExo70J proteins share a highly similar N-terminal domain that is present only in proteins from legumes (Table 1; Fig. 4). BLAST searches of proteomes of non-plant organisms revealed that the closest homologues of the N-terminal domains are eukaryotic cytochrome b561-like proteins from bird species, despite the fact that plants including soybean also contain this family of proteins (Supplementary Figure S4). Furthermore, a number of online transmembrane prediction algorithm servers revealed that the N-terminal domains of GmWRP1 and some of the GmExo70J proteins contain five-pass TM helices (Fig. 7), similar to eukaryotic cyt b561 proteins.

Eukaryotic cyt b561 proteins are integral membrane proteins usually containing five to six alpha-helical transmembrane segments (Lu *et al.*, 2014). They are secretory vesicle-specific electron transport proteins that bind two haem groups non-covalently and play critical roles in ascorbate homeostasis and iron reduction. For example, in vertebrates, cyt b561 proteins might act as a ferric-chelate reductase, catalysing the reduction of Fe^3+^ to Fe^2+^, which is associated with the transport of iron from the endosome to the cytoplasm (Asard *et al.*, 2013). It is assumed that this protein uses ascorbate as the electron donor (Asard *et al.*, 2013). Alignment of the soybean domains with the consensus cyt b651 sequences revealed that only two of the four haem-binding histidine residues highly conserved in cyt b651 proteins are still present in the legume-specific TM domains (Supplementary Figure S4). Therefore, whether the TM domains in GmWRP1 and GmExo70J proteins act as cyt b651 proteins in legumes remains to be determined.

### The N-terminal TM domains of GmWRP1 and GmExo70JX are targeted to the Golgi apparatus

**Fig. 8.**
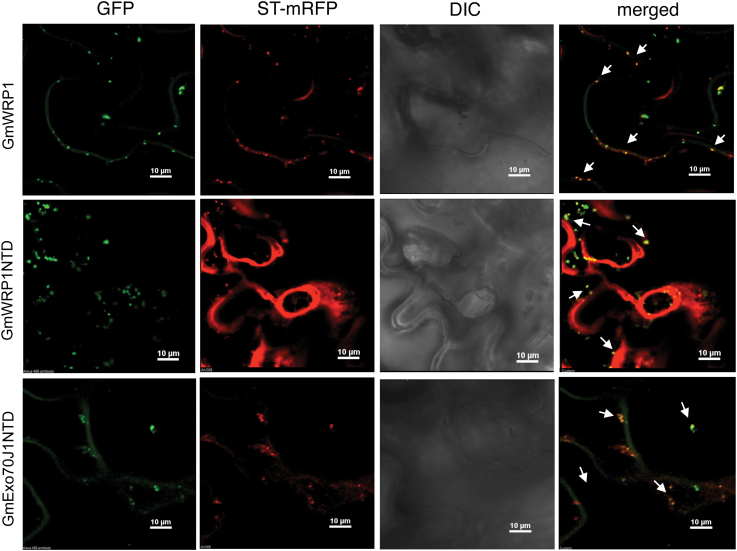
The N-terminal TM domains are sufficient for Golgi apparatus localization. GFP fusion proteins for GmWRP1 and the N-terminal TM domains (NTDs) of GmWRP1 (GmWRP1NTD) and GmExo70J1 (GmExo70J1NTD) were co-expressed with the Golgi apparatus marker gene ST-RFP in *N. benthamiana*. A majority of GFP punctate fluorescence signals from GmWRP1, GmWRP1NTD, and GmExo70J1NTD were also labelled by the ST-mRFP Golgi marker signals, and some are indicated by arrows. Co-expression of GmWRP1NTD–GFP also altered the labelling patterns of the ST-RFP Golgi marker. Bar, 10 µm.

GmWRP1 and seven GmExo70J proteins share a highly similar N-terminal TM domain (Fig. 4) and are localized to the Golgi complex (Figs 3 and 6), suggesting that the conserved N-terminal domains in these proteins may function as Golgi-targeting domains. To test this possibility, we fused the N-terminal TM domains of GmWRP1 (GmWRP1NTD) and GmExo70J1 (GmExo70J1NTD) with GFP (Supplementary Figure S1) and transiently co-expressed the GFP fusion constructs with the ST-mRFP Golgi marker in *N. benthamiana*. As a control, we also included GmWRP1–GFP co-expressed with ST-mRFP Golgi marker in the experiments. As expected, expression of GmWRP1–GFP in tobacco leaf epidermal cells produced punctate fluorescent signals that were mostly labelled by the ST-mRFP Golgi marker (Fig. 8). Likewise, expression of GmWRP1NTD–GFP led to the formation of punctate fluorescent signals in tobacco leaf epidermal cells that were also mostly labelled by the ST-RFP Golgi marker. Interestingly, unlike in the cells expressing GmWRP1–GFP, where the fluorescent signals from the ST-RFP Golgi marker were largely punctate, co-expression of GmWRP1NTD–GFP led to the formation of not only punctate fluorescent signals but also an extensive network, particularly around certain unknown subcellular structures or organelles as revealed by differential interference contrast (DIC) images (Fig. 8). When the GmExo70J1NTD–GFP fused gene was expressed in *N. benthamiana*, the levels of fluorescent signals were again lower than those of GmWRP1–GFP but higher than those of GmExo70J1–GFP (Supplementary Figure S3). The GmExo70J1NTD–GFP fusion proteins generated largely punctate fluorescent signals that were again largely labelled by the ST-RFP marker (Fig. 8). When the GmExo70J1NTD–GFP construct was co-expressed with the Syp41-mCherry TGN marker or ARA6-m-Cherry MVB marker, there was little overlap between the punctate GFP and mCherry signals (Supplementary Figure S5). Thus, the N-terminal regions of GmWRP1 and GmExo70J1, which contain a cyt-b651-like TM domain, are sufficient for targeting to the Golgi apparatus. Furthermore, fluorescent patterns of the ST-mRFP maker were apparently altered by coexpressed GmWRP1NTD–GFP and some of the GmExo70J–GFP fusion proteins because no such changes in the fluorescent signal patterns of the Golgi marker were observed when it was expressed alone (Supplemental Fig. 6A) or when it was co-expressed with the full-length GmWRP1–GFP protein (Fig. 3B).

To further examine the role of the N-terminal TM domain, we generated two additional GFP fusion proteins and examined their subcellular localization. In the first construct, we deleted the N-terminal TM domain of GmWRP1 and fused the remaining C-terminal domain (CTD) to generate the GmWRP1CTD–GFP construct (Supplementary Figure S1). We also placed the GFP tag to the C-terminus of GmExo70J3, which does not contain the legume-specific TM domain at its N-terminus (Table 1; Supplementary Figure S1). When the two constructs were expressed in tobacco cells, we observed largely dispersed fluorescence signals with few punctate structures not only in the cytoplasm but also in the nucleus of some cells (Supplementary Figure S6B). These results further indicate that the N-terminal TM domains in the GmWRP1 and GmExo70J proteins are necessary for targeting to the Golgi complexes.

### Expression of *GmWRP1* and *GmExo70* genes

**Fig. 9.**
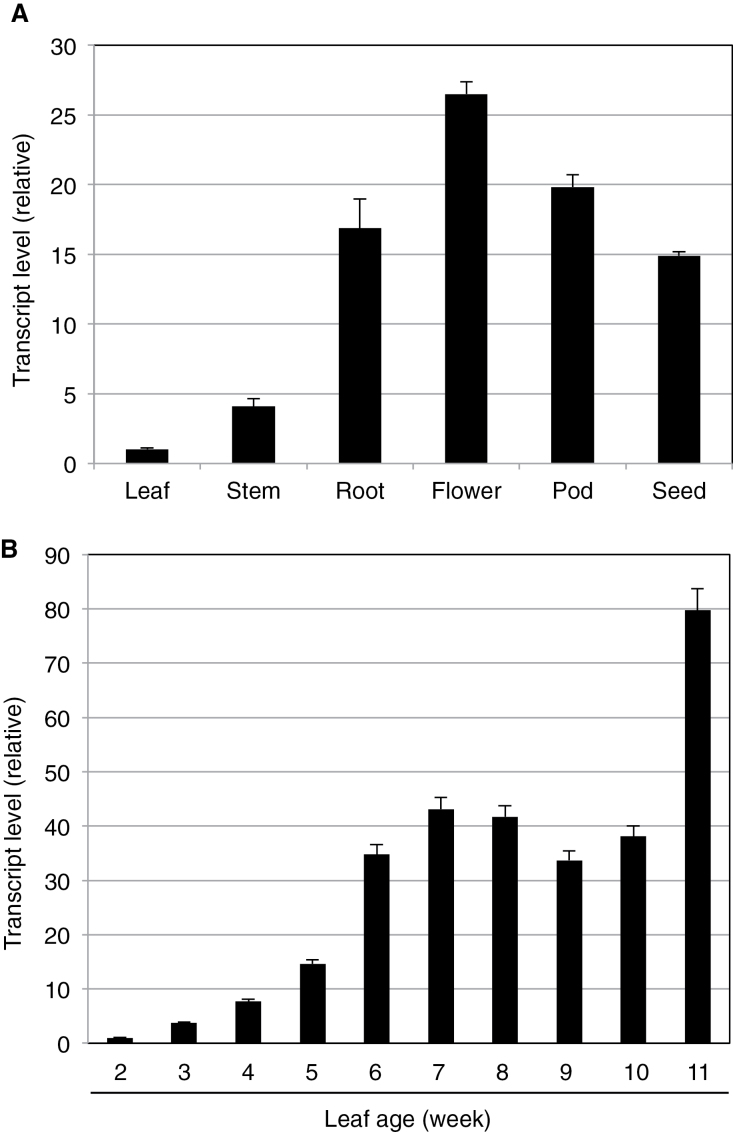
Expression analysis of *GmWRP1*. (A) Expression of *GmWRP1* in different plant tissues. Young leaves, stems, and roots were collected from 4-week-old seedlings at vegetative (V) 2 stage; blooming flowers were sampled from plants at reproductive (R) 2 stage; and pods and seeds were collected at R3 and R7 stages, respectively. (B) Age-regulated expression of *GmWRP1* in soybean leaves. The first trifoliolate leaves were collected from 2-week-old seedlings (V1 stage) to 11-week-old plants (R7 stage) at 1-week intervals. *GmWRP1* expression was analysed by quantitative qRT-PCR using a soybean actin gene as an internal control.

**Fig. 10.**
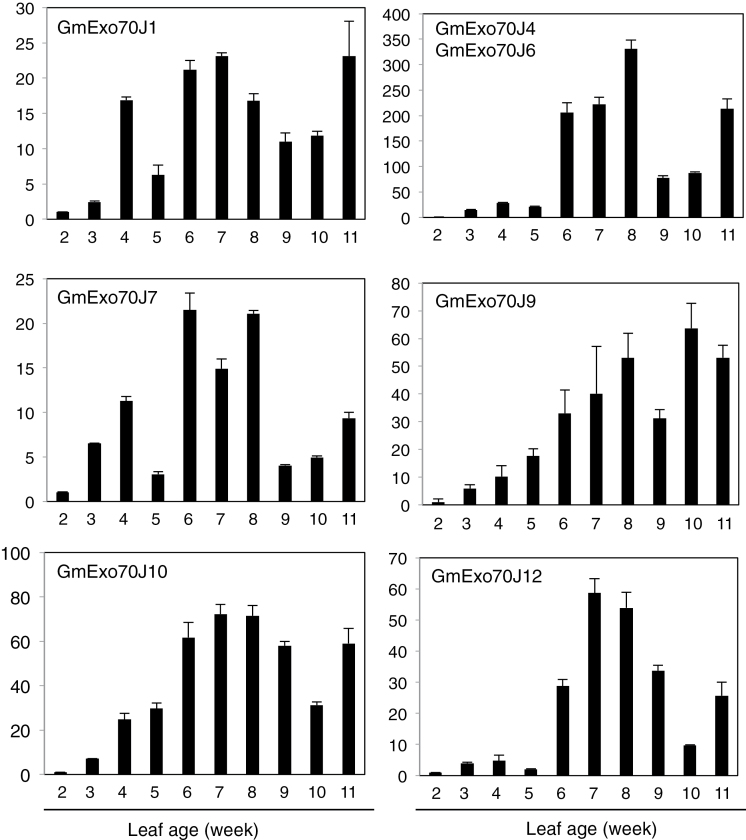
Age-regulated expression of *GmExo70J* genes in soybean leaves. The first trifoliolate leaves were collected from 2-week-old seedlings at vegetative (V) 1 stage to 11-week-old plants at reproductive (R) 7 stage at 1-week intervals. *GmExo70J* gene expression was analysed by quantitative qRT-PCR using a soybean actin gene as an internal control.

To further characterize GmWRP1 and related GmExo70J proteins with the novel N-terminal TM domains, we examined their expression in soybean using qRT-PCR. First, we examined the transcript levels of *GmWRP1* in different soybean tissues. As shown in Fig. 9A, expression of *GmWRP1* was detected in all examined tissues but the expression levels in roots, flowers, pods, and seeds were substantially higher than those in young leaves and stem. In leaves, the expression of the gene appeared to be developmentally regulated. At young ages (1-week old), the expression of the gene was very low but could steadily increase with increasing leaf ages (Fig. 9B). In the 9–10-week-old leaves, the expression levels were ~50–80 times higher than those in the 1-week-old leaves (Fig. 9B). Age-regulated expression was also observed with the seven GmExo70J proteins with the N-terminal TM domain. As shown in Fig. 10, transcripts for the *GmExo70J* genes were generally elevated with increased age in leaves, although the magnitude and kinetics of the increase varied among the *GmExo70J* genes.

## Discussion

### Origin and evolution of legume-specific *WRP1* and *Exo70J* genes

In the present study, we have identified a novel WRKY-related protein and a group of Exo70 proteins containing a novel Golgi-targeting TM domain that is apparently present only in proteins from legumes (Fig. 1). Because of a lack of close homologues in non-legume plants, the evolutionary origin of the TM domain in these legume proteins is unclear. Intriguingly, BLAST searching of proteomes of plant and non-plant organisms revealed that the closest homologues of the TM domains are endosomal cyt b561-like proteins from birds (Supplementary Figure S4). The fact that the TM domains are structurally more similar to bird than plant cyt b561 proteins raises the intriguing possibility that the legume-specific TM domains may have been derived from horizontal gene transfer from birds. Since close structural homologues of GmWRP1 and GmEx070J proteins are present in other legumes, including *Medicago*, common bean, and chickbean, but not in non-legume plants, the appearance of the novel TM domain must have predated the speciation of legumes, which is widely believed to be about 40–50 million years ago. This interpretation is consistent with the observation that the newly identified *GmExo70J* genes with the novel TM domains are distributed on four chromosomes of soybean, an ancient tetraploid with two genome duplications that occurred at approximately 59 and 13 million years ago (Schmutz *et al.*, 2010). *GmExo70J* genes on Chr 2, 6, and 14 have further expanded through local duplication that generated clusters of *Exo70J* genes. Interestingly, unlike the *Exo70J* gene family, there is only a single *GmWRP1* gene in the soybean genome, and it is within a cluster of *GmExo70J* genes on Chr 14. However, there are two close homologues of *GmWRP1*, also within the clusters of *Exo70J* genes on two separate chromosomes in the genome of common bean, which is a diploid. Therefore, it is likely that the novel N-terminal TM domains from the *WRP1* and *Exo70J* genes evolved from a common ancestor.

Those GmExo70 proteins containing the novel Golgi-targeting TM domain belong to an Exo70 subclade phylogenetically distinct from the nine Exo70 subclades previously identified in plants (Fig. 5; Supplementary Figure S2) (Synek *et al.*, 2006; Chong *et al.*, 2010). This distinct Exo70J subclade contains at least 12 members in soybean, and some of these GmExo70J proteins do not contain the novel N-terminal TM domains (Table 1). Phylogenetic analysis indicates that the GmExo70J proteins are most closely related to two GmExo70B proteins (Glyma02g39790 and Glyma14g37840), which are encoded by two genes also within the *GmExo70J* genes clusters on Chr 2 and 14, respectively (Fig. 5). These observations suggest a possible evolutionary history of the GmExo70J proteins that started with the duplication of an *Exo70B* gene. Diversification of the duplicated *Ex70B* genes would then lead to the birth of an ancestral *Exo70J* gene, whose subsequent duplication resulted in additional *Exo70J* genes. At least one of *Exo70J* genes apparently underwent further diversification through fusion with a cyt b561-like TM sequence followed by additional duplications. Alternatively, fusion with the cyt b561-like TM sequence might have occurred to the ancestral *Exo70J* gene prior to its duplication, but during the history of subsequent proliferation and diversification some members of the *Exo70J* gene subfamily might have lost the N-terminal cyt b561-like sequences.

### Cellular roles of legume-specific WRP1 and Exo70J proteins

We have shown that GmWRP1 and GmExo70J containing the N-terminal TM domain are localized to the Golgi apparatus (Figs 3 and 6). Further analysis with the TM–GFP fusion further indicated that the N-terminal TM domain is sufficient for targeting fused GFP to the Golgi complex (Fig. 8). Thus, fusion of the novel TM domain with a WRKY or Exo70 protein could serve to generate new biological functions of the fused protein through altering their subcellular localization. As transcription factors, WRKY proteins are usually localized in the nucleus. Exo70 proteins from yeast and animals are often localized on plasma membrane that tethers Golgi-derived vesicles to it during exocytosis (Finger *et al.*, 1998; Guo *et al*., 1999*a*, *b*; Boyd *et al.*, 2004; Samuel *et al.*, 2009). Unlike yeast and animal Exo70 proteins, however, Exo70 proteins have expanded dramatically in plants and have undoubtedly diversified in biological functions through a variety of means. For example, studies of a number of *Arabidopsis* Exo70 proteins have revealed their important roles not only in exocytosis but also in other cellular processes including fusion of autophagosomes with vacuoles (Kulich *et al.*, 2013). The diverse roles of plant Exo70 proteins are likely to be associated with its various subcellular localization patterns, ranging from the cytosol to various endosomal compartments, as shown experimentally (Chong *et al.*, 2010; Drdova *et al.*, 2013; Kulich *et al.*, 2013; Ding *et al.*, 2014). How plant Exo70 proteins are targeted to different subcellular compartments will be of great interest for understanding the cellular basis of their biological functions, and information about the Golgi-targeting determinants of some of the soybean Exo70J proteins could provide useful clues for addressing this important question.

The N-terminal TM domains from GmWRP1 and some of the GmExo70J proteins are more than 150 amino acid residues in length and, therefore, may have functions in addition to Golgi targeting. Overexpression of the N-terminal TM domain of GmWRP1, but not GmExo70J1, in tobacco cells led to an alteration of the distribution of the ST-mRFP Golgi marker (Fig. 8), which is probably a result of altered structures, movement, or other important properties of the Golgi complex. In yeast and animal cells, drastically altered Golgi structures have also been observed and in many cases attributed to Golgi fragmentation, which produces Golgi fragments that can coalesce with each other or combine with other cellular molecules to generate discrete cellular structures (Gonatas *et al.*, 2006; Bellouze *et al.*, 2014; Petrosyan and Cheng, 2014). The strong effect on an important subcellular compartment by expressing some GmExo70J proteins and the truncated GmWRP1NTD protein could be explained by a dominant negative activity of the N-terminal TM domain through, for example, interactions with proteins important for biogenesis and trafficking of the Golgi complex. The N-terminal domains are predicted to contain five transmembrane helices that could form complexes with other integral transmembrane proteins in the Golgi complex. The spacers between the five transmembrane helices could also interact with proteins in the cytosol or Golgi lumen. In addition, as homologues of eukaryotic cyt b561 proteins, the legume-specific N-terminal TM domain could have a secretory, vesicle-specific electron transport function. In vertebrates, cyt b561 proteins can act as ferric-chelate reductases, catalysing the reduction of Fe^3+^ to Fe^2+^, which is associated with the transport of iron from the endosome to the cytoplasm. Alignment of the soybean TM domains with the consensus cyt b651 sequences revealed that only two of the four haem-binding histidine residues highly conserved in cyt b651 proteins are still present in the legume-specific TM domains. Therefore, it will be of great interest to determine whether the legume-specific TM domains still bind haem and, if so, act as cyt b651 proteins.

### Biological functions of legume-specific WRP1 and Exo70J proteins

In contrast to a single gene in yeast and many animals, plants contain a large number of Exo70 proteins in their genomes. Based on analysis of their expression patterns, subcellular localization, and mutants, there is compelling evidence that plant Exo70 proteins play critical roles in a broad range of biological processes (Zarsky *et al.*, 2013). *Arabidopsis* Exo70A1 functions in vesicle trafficking important for cell/organ morphogenesis, secondary cell wall thickening in tracheary elements, localized deposition of cell wall pectin, and pollen–stigma compatibility (Samuel *et al.*, 2009; Kulich *et al.*, 2010; Li *et al.*, 2013; Safavian *et al.*, 2014). Other Exo70 proteins participate in vesicle trafficking in plant–pathogen interactions and in transport of autophagosomes to the vacuole (Stegmann *et al.*, 2012; Kulich *et al.*, 2013; Stegmann *et al.*, 2013). *Arabidopsis* Exo70E2 is required for recruitment of exocyst subunit recruitment to a novel double-membrane organelle termed the exocyst-positive organelle (EXPO) that may mediate unconventional protein secretion in plant cells (Ding *et al.*, 2014). As legume-specific proteins, the biological functions of the Exo70J proteins are likely to be associated with the cellular processes uniquely important to legumes, such as their association with symbiotic *Rhizobia* for nitrogen fixation. During the complex legume–rhizobium association, the rhizobial bacteria invade plant roots and induce nodules in which the bacteria reduce atmospheric nitrogen to ammonia using energy provided by plant cells and supply the plant with nitrogenous compounds (Udvardi and Poole, 2013). Exo70J-mediated vesicle trafficking could play roles during the legume–rhizobium association. It is also worth noting that both *Arabidopsis* AtExo70B proteins (AtExo70B1 and AtExo70B2) have been shown to play roles in plant immune responses to microbial pathogens (Pecenkova *et al.*, 2011; Kulich *et al.*, 2013; Stegmann *et al.*, 2013). *Arabidopsis* Exo70B1 is also involved in autophagy-related membrane traffic to the vacuole (Kulich *et al.*, 2013), a process implicated in a range of plant processes including immune responses (Zhou *et al.*, 2014). As legume-specific Exo70J proteins are evolutionarily closely related to Exo70B proteins (Fig. 5), it is tempting to speculate that some legume Exo70J proteins might be involved in the communication between legumes and rhizobial bacteria.

GmExo70J-mediated vesicle trafficking could also be involved in the uptake, secretion, and transport of nitrogen, nitrogen-containing compounds, and photosynthates, which occurs not only in legume nodules but also throughout the whole legume plants (Udvardi and Poole, 2013). Indeed, expression of the *GmWRP1* and *GmExo70J* genes was detected throughout the plants (Figs 9 and 10). Furthermore, induced expression of these genes in leaves with increased ages may suggest a possible role in the regulation of leaf senescence. Plant senescence is a developmental process that serves, among other functions, as a process for nutrient redistribution. In the older parts of plants such as lower leaves, several proteolytic pathways including autophagy participate in the degradation of defective and unneeded cellular structures and molecules, including chloroplasts and chloroplast proteins, for efficient nutrient re-localization and utilization by young tissues and developing fruits and seeds (Hanaoka *et al.*, 2002; Bassham, 2007; Yoshimoto *et al.*, 2009). In autophagy-deficient mutants, lack of degradation of the defective and unneeded cellular structures and molecules can presumably lead to cellular stress, and accumulation of reactive oxygen species and salicylic acid, which could operate through a positive feedback loop to trigger early senescence and programmed cell death (Yoshimoto *et al.*, 2009). The GmWRP1 and GmExo70J proteins could play a role in the regulation of senescence of soybean leaves through modification and transport of leaf proteins. Further studies of the unique Exo70 proteins through analysis of their associated exocyst complex subunits, tissue-specific expression patterns, dynamic subcellular localization and, most importantly, isolation and characterization of mutants through, for example, gene editing technology, will be necessary to fully understand the unique roles of the subfamily in legumes.

## Supplementary material

Supplementary data can be found at *JXB* online.

Supplementary Table S1. Primers for generating GFP fusion constructs.

Supplementary Table S2. Primers for qRT-PCR.

Supplementary Table S3. Primers for generating BPMV silencing constructs.

Supplemental Figure. S1. Diagram of GmWRP1 and GmExo70J proteins and their GFP fusions.

Supplementary Figure S2. Phylogenetic analysis of plant Exo70 domains.

Supplementary Figure S3. Western blot analysis of GmWRP1– and GmExo70J–GFP fusion proteins expressed in tobacco leaves.

Supplementary Figure S4. Sequence comparison of the N-terminal TM domains of GmWRP1 and cyts b561 from bird species (*Ficedula albicollis*, *Taeniopygia guttata*, and *Pseudopodoces humilis*) and soybean.

Supplementary Figure S5. Lack of co-localization of GmExo70J1NTD with TGN marker Syp41 or MVB marker ARA6.

Supplementary Figure S6. Subcellular localization of ST-mRFP, and GmWRP1CTD– and GmExo70J3–GFP.

## Funding

This work was supported by the China Ministry of Agriculture Transgenic Crop Major Project (grant no. 2012ZX08009004), the China Postdoctoral Science Foundation (grant no. 2013M531466), and the US National Science Foundation (grant no. IOS-0958066).

## Supplementary Material

Supplementary Data
