# Supplementary material for: Identification and characterization of a novel group of legume-specific, Golgi apparatus-localized WRKY and Exo70 proteins from soybean
Source: J Exp Bot. 2015 Mar 24;66(11):3055–70. doi: 10.1093/jxb/erv104 (PMC4449531; doi:10.1093/jxb/erv104)
Supplement: Supplementary Data [file supp_66_11_3055__index.html]

Identification and characterization of a novel group of legume-specific, Golgi apparatus-localized WRKY and Exo70 proteins from soybean — Supplementary Data 

# Identification and characterization of a novel group of legume-specific, Golgi apparatus-localized WRKY and Exo70 proteins from soybean

## Supplementary Data

Data files

**Files in this Data Supplement:**

- Supplementary Data - Supplementary Data
