# Supplementary material for: Identification and characterization of a novel group of legume-specific, Golgi apparatus-localized WRKY and Exo70 proteins from soybean
Source: J Exp Bot. 2015 Mar 24;66(11):3055–70. doi: 10.1093/jxb/erv104 (PMC4449531; doi:10.1093/jxb/erv104)
Supplement: Supplementary Data [file supp_erv104_jexbot137539_file001.pdf]

Supplemental Figure S1

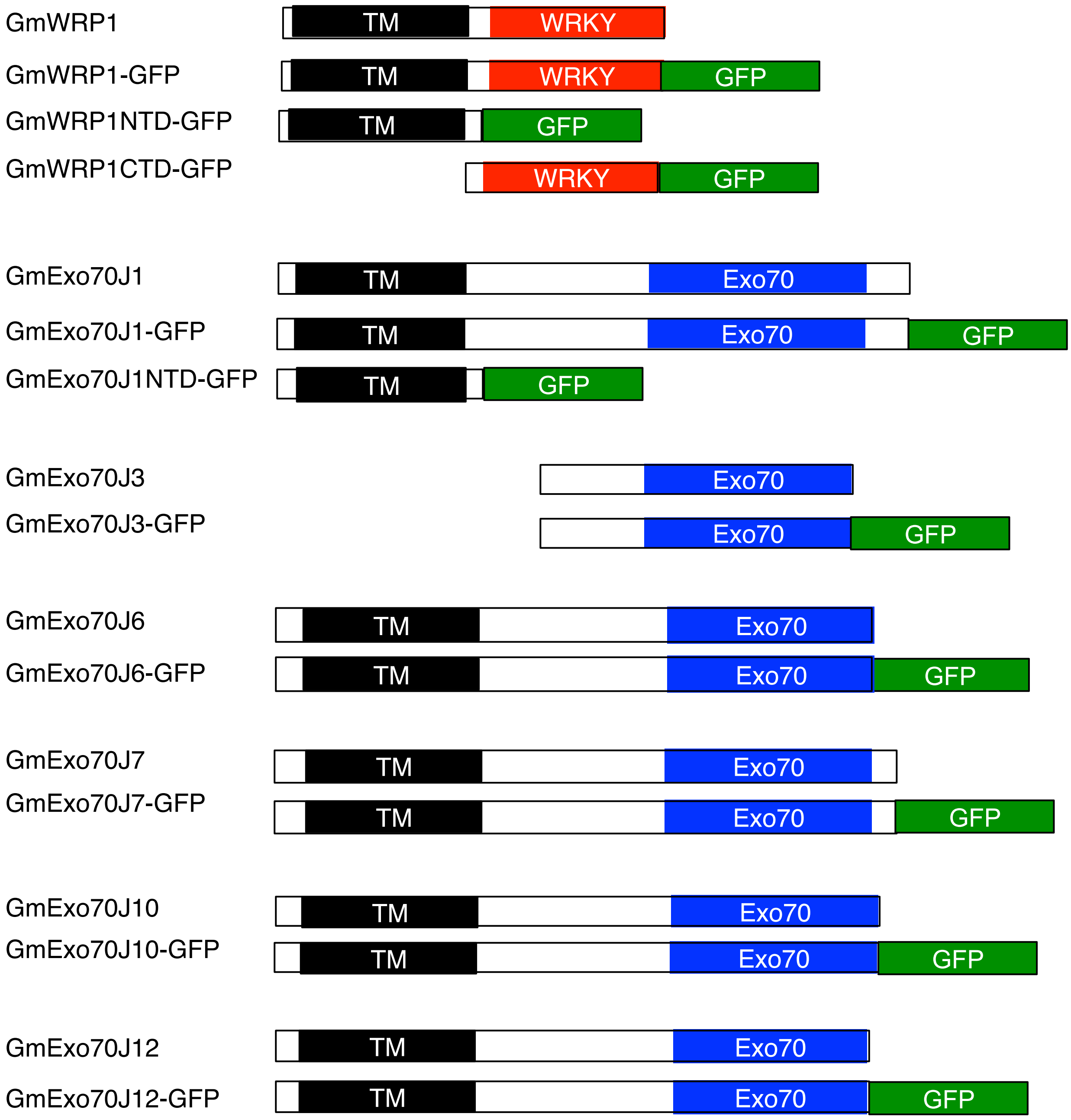

Supplemental Figure S1. Diagram of GmWRP1 and GmExo70J proteins and their GFP fusions.

Supplemental Figure S2

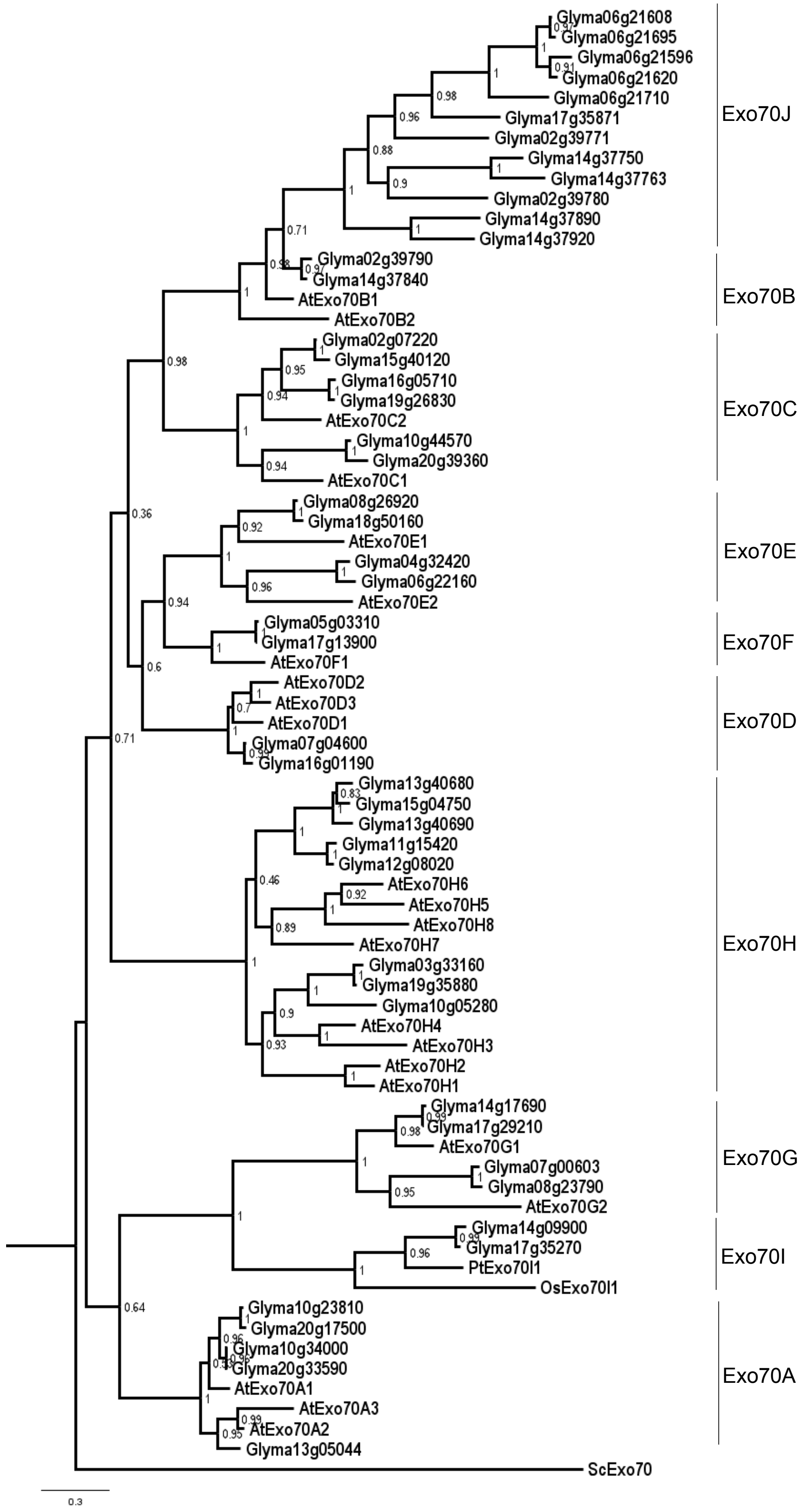

Supplemental Figure S2. Phylogenetic analysis of plant Exo70 domains. The phylogenetic tree was inferred using the maximum likelihood methods from 47 soybean GmExo70, 23 Arabidopsis AtExo70, two Exo70I protein from rice and poplar and yeast Exo70. Bootstrap values from 1,000 replicates were used to assess the robustness of the tree. Ten subclades of plant Exo70 proteins are indicated.

Supplemental Figure S3

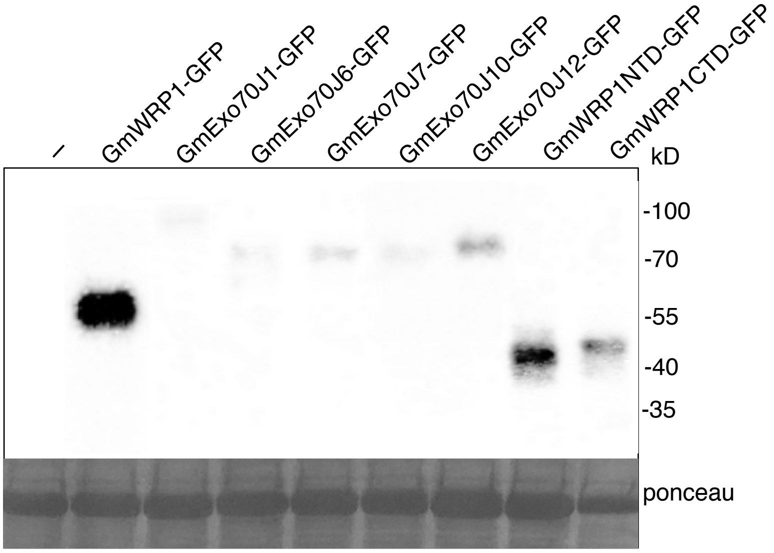

Supplemental Figure S3. Western blot analysis of GmWRP1- and GmExo70J-GFP fusion proteins expressed in tobacco leaves. Total proteins were isolated from infiltrated tobacco leaves and separated by SDS-PAGE. The GFP fusion proteins were detected with an anti-GFP antibody. Ponceau staining of the blot was also shown as loading control.

Supplemental Figure S4

```
GmWRP1         17   IVGFLSSVIGLIC---YALSSSFNHLFG--EWNFLKIILYAVISFSISSIMLLLKK----W  68
FAcytb561      16   LVGFLSVILSLVWVFHYREGLSWDGSLG--EFNWHPVLIITGFVF-IQGIAIIVYRLPWTW  73
TGcytb561      16   LVGFVSVILSLVWVFHYREGLSWDGSLG--EFNWHPVLIITGFVF-IQGIAIIVYRLPWTW  73
PHcytb561      16   LVGFVSVILSLVWVFHYREGLSWDGSPG--EFNWHPVLIITGFVF-IQGIAIIVYRLPWTW  73
Glyma07g27950  12   LFGILAFILLLVWLLHYREGIDYDSDNGFRVFNAHPLLMYSCFIF-LAGEAMMVFQ---TI  68
Glyma19g05890  18   AVGVAVTVLLLTWAVHFRGGLALVSDNKDLIFNVHPVLMVIGLVL-INGEGMLAYK---TL  74
Glyma20g01530  12   LFGILAFILLLVWLLHYREGIDYDSDKGFRVFNAHPLLMYSCFIF-LAGEEV---------  62
                                                      *

GmWRP1         69   KLSKSFMLKAHVGV----LVLLITSVYSFVSDKAVNGKPDMLSLISCFAFAFMSLCLSKQI  125
FAcytb561      74   KCSKLLMKFIHAGLNTIAMILAIVSMVAVFEFHNAKNIPNMYSLHSWIGLAAV-IFYSLQL  133
TGcytb561      74   KCSKLLMKFIHAGLNTIAMILAIVSMVAVFEFHNAKNIPNMYSLHSWIGLTAV-IFYSLQL  133
PHcytb561      74   KCSKLLMKFIHAGLNTIAMILAIVSMVAVFEFHNAKNIPNMYSLHSWIGLTAV-IFYSLQL  133
Glyma07g27950  69   PSERKVKKFVHMTLHLIAIVLGIVGLNAVFKFHDMQNIPNVYSLHSWIGIGTF-CLFALQW  128
Glyma19g05890  75   SGTKNFKKSVHLALQFAALILSLIGVWAAWKFHIDKGIDNFYSLHSWLGLACL-FLFTIQW  134
Glyma20g01530  63   -CS-------HA-LHLIAIVLGIVGLNAVFKFHGMENIPNVYSLHSWIGIVTF-CLFGLQW  113
                              *                                  *

GmWRP1         126  DLGFGADLLNFFLGCLTVQLMHIHLM--LSIVAAIFCYCFMFFRSKL  170
FAcytb561      134  FLGFAVFLLPFAPVHLRVALMPIHVYSGLTIFATVIATALMGITEKL  180
TGcytb561      134  FLGFAVFLLPFAPVHLRVALMPIHVYSGLTIFATVIATALMGITEKL  180
PHcytb561      134  FLGFAVFLLPFAPVHLRVALMPIHVYSGLTIFATVIATALMGITEKL  180
Glyma07g27950  129  LFGFVVFMLQGASATTRAKVLPWHKVGGRALLFMAVCAAETGLMEKS  175
Glyma19g05890  135  GAGFATFWYPGGSRNSRAALLPWHVFFGIYIYCLAIATTATGILEKA  181
Glyma20g01530  114  LFGFVVFMLQGASVNTRAKVLPWHKVGGRALLFMAVCAAETGLMEKS  160
                                           *
```

Supplemental Figure S4. Sequence comparison of the N-terminal TM domains of GmWRP1 and cyts b561 from birds and soybean. Cyts b561-like proteins from bird species *Ficedula albicollis* (FAcytb561, XP 005049393.1), *Taeniopygia guttata* (TGcytb561, XP 002198627.1), *Pseudopodoces humilis* (PHcytb561, XP 005519846.1) and three soybean cyt b561-like proteins are aligned and the amino acid residues in the cyt b561-like proteins identical to those of the TM domain of GmWRP1 are in red. The four conserved heme-binding histidin residues in the cyts b561 proteins are in green and indicated with asterisks.

Supplemental Figure S5

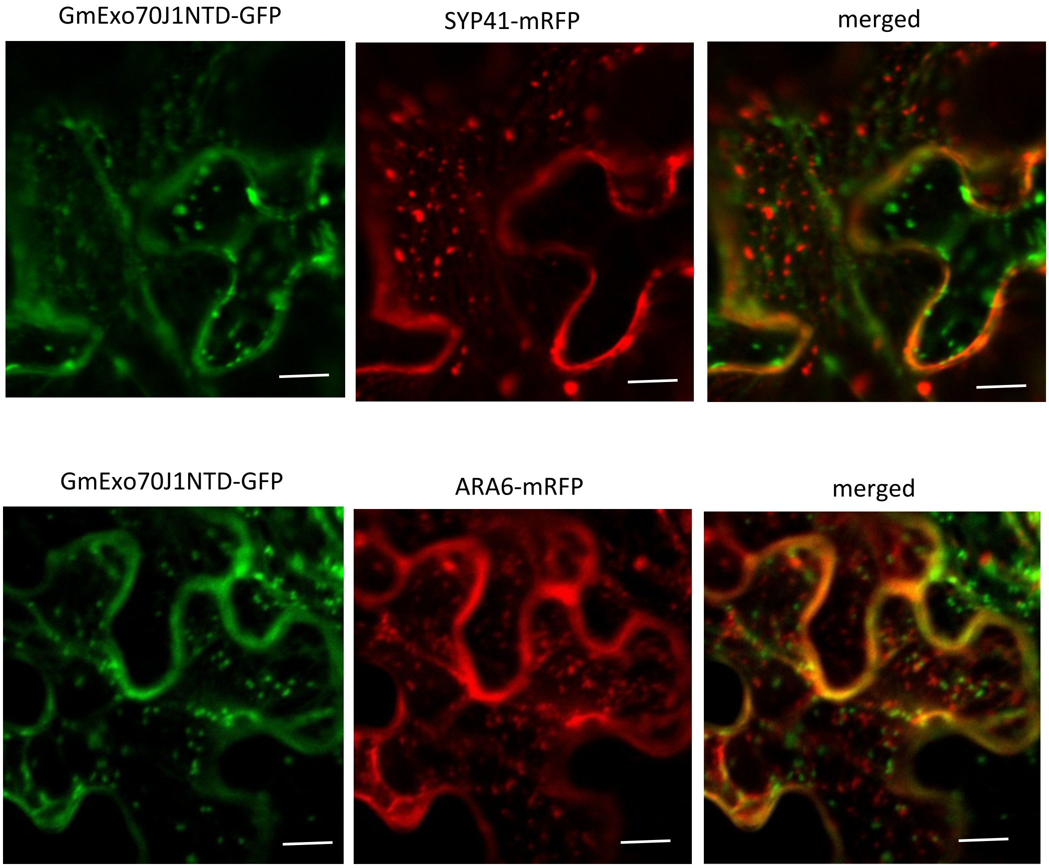

Supplemental Figure S5. Lack of colocalization of GmExo70J1NTD with TGN marker Syp41 or MVB marker ARA6.

The GmExo70J1NTD-GFP fusion gene was coexpressed with the TGN marker Syp41-mCherry or the MVB marker gene ARA6-mCherry in *N. benthamiana*. Few GmExo70J1NTD-GFP fluorescence signals were labeled by punctate Syp41- or ARA6-mCherry signals. Bar=10 μm.

Supplemental Figure S6

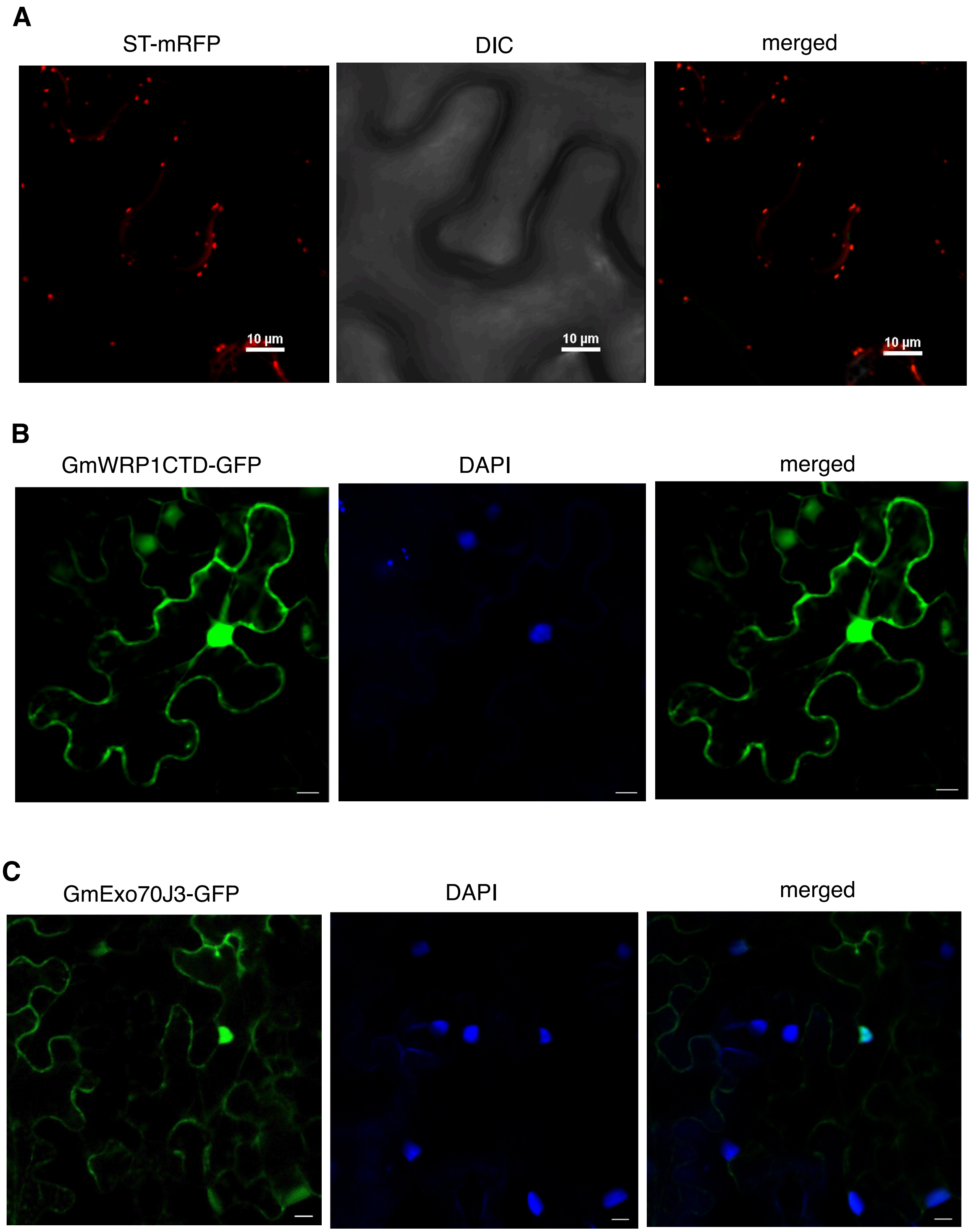

Supplemental Figure S6. Subcellular localization of ST-mRFP, GmWRP1CTD- and GmExo70J3-GFP.

A. The red fluorescent signal, DIC and merged images of tobacco cells expressing the ST-mRFP Golgi marker alone.

B. The GFP signals, DAPI staining and merged images of tobacco cells expressing the GmWRP1CTD- GFP fusion gene.

C. The GFP signals, DAPI staining and merged images of tobacco cells expressing the GmExo70J3-GFP fusion gene.

Bar=10 μm.

Supplemental Table S1. Primers for generating GFP fusion constructs

Name	Gene identifier	Primers
GmWRP1	Glyma14g37960	F: AGCCTCGAGATGCCTTTCCGAAATTCCAATAT<br>R: AGCGGATCCTATTTCATTTGGATATTGAGGAGTTC
GmWRP1NTD	Glyma14g37960	F: AGCCTCGAGATGCCTTTCCGAAATTCCAA<br>R: AGCGGATCCGTCATCAAACTCTCTTTTTCCATCT
GmWRP1CTD	Glyma14g37960	F: AGCCTCGAGATGTTTTTTCGTTCCAAGTTGG<br>R: AGCGGATCCTATTTCATTTGGATATTGAGGAGTTC
GmExo70J1	Glyma02g39771	F: AGCGTCGACATGCTCATTGAGATTCAG<br>R: AGCCCCGGGTCCCTTACAAGAAA
GmExo70J1NTD	Glyma02g39771	F: AGCCTCGAGATGCTCATTGAGATTCAGAGTTG<br>R: AGCGGATCCACTGCTATTGATTTCTGATGAAAGTG
GmExo70J3	Glyma06g21596	F: AGCCTCGAG ATGGGTTGCATAGAGG<br>R: AGCGGATCC CTCTCTTACAAGAAAC
GmExo70J6	Glyma06g21695	F: AGCCTCGAGATGGTCTTCATGTTGATCATGATC<br>R: AGCGGATCCCTCTCTTACAAGAAACAAATTGTTGA
GmExo70J7	Glyma06g21710	F: AGCCTCGAGATGGTCTTCATGTTGATCATGATCA<br>R: AGCGGATCCCATTCTTACAAGAAACAAATTATTGAG
GmExo70J9	Glyma14g37763	F: AGCCTCGAGATGGCGGCTCTCTTTGTTG<br>R: AGCAGATCTTTTGATCCTTGAGGCCTGTG
GmExo70J12	Glyma17g35871	F: AGCCTCGAGATGCTCATAATAAAGATTCGGAGT<br>R: AGCCCATGGCCTCTAGTTCTTTCTCCCATACAAGA

Supplemental Table S2. Primers for qRT-PCT

Name	Gene identifier	Primers
GmWRP1	Glyma14g37960	F: ACAAATGCACACAACCCACT<br>R: AGGTGACAGGCTCTGAAGGT
GmExo70J1	Glyma02g39771	F: AGCCAGAATAGCAAGGCTGT<br>R: TAGCCAATAGGTCCCAATCC
GmExo70J4	Glyma06g21608	F: GTCTTGTCCAGACTCCAGTG<br>R: TCCTCCTTTAACCCTGCTGT
GmExo70J6	Glyma06g21695	F: GTCTTGTCCAGACTCCAGTG<br>R: TCCTCCTTTAACCCTGCTGT
GmExo70J7	Glyma06g21710	F: TCCTTCAATGAACACCTGGA<br>R: CTGCAATCTCGCAATGAAGT
GmExo70J9	Glyma14g37763	F: GTGGAGGGCTTCATCTGATT<br>R: AGTCGTCTCTGGACTTGGCT
GmExo70J10	Glyma14g37890	F: GTGAGCGACGACTCTGTGATA<br>R: AAATGTCCCTGCTTGCTTCT
GmExo70J12	Glyma17g35871	F: TCCCTCTTGAATTCCCAGTC<br>R: TGGTCCTCGTTGACAACATT

Supplemental Table S3. Primers for BPMV silencing constructs

Name	Gene identifier	Primers
GmWRP1	Glyma14g37960	F: AAGGGATCCTCTATTGTTGCAGCCATATTTTG<br>R: TTGGGTACCTCTTCACATAGCAAGTGGGTTG
GmExo70J1	Glyma02g39771	F: AAGGGATCCTCGATAAAAGCTTTGAAT<br>R: TTGGGTACCCTTGCACAGAAGGTGAAAG
GmExo70J4	Glyma06g21608	F: AAGGGATCCATAGCCATGATAACAAACCTG<br>R: TTGGGTACCCTCTTACAAGAAATAAATTGT
GmExo70J6	Glyma06g21695	F: AAGGGATCCTTCGGGGAATTGTTAAGT<br>R: TTGGGTACCCCCATGAGCTTCTCAGAT
GmExo70J7	Glyma06g21710	F: AAGGGATCCAGAAACAGAACCAGTCCT<br>R: TTGGGTACCCCATGTTGAGCTCTTCCAAT
GmExo70J9	Glyma14g37763	F: AAGGGATCCAAGGGATTGACGATTCGT<br>R: TTGGGTACCCTTCCAAAATGCTCTCTAG
GmExo70J10	Glyma14g37890	F: AAGGGATCCCAATTGCTGAATATTGCT<br>R: TTGGGTACCCAGATGTTCCAGCTCCA
GmExo70J12	Glyma17g35871	F: AAGGGATCCGACATTTTGAAGGTGGACAT<br>R: TTGGGTACCCCTCCCATACAAGAAACAAAT
